# What is the effectiveness of various invitation methods to a colonoscopy in the early detection and prevention of colorectal cancer? Protocol of a systematic review

**DOI:** 10.1186/s13643-020-01312-x

**Published:** 2020-03-06

**Authors:** Undine Antonia Stark, Thomas Frese, Susanne Unverzagt, Alexander Bauer

**Affiliations:** 1 Institute of General Practice and Family Medicine, Medical Faculty, Martin-Luther-University Halle-Wittenberg, Halle, Germany; 2 Department of General Practice, Medical Faculty, University of Leipzig, Leipzig, Germany

**Keywords:** Colorectal cancer, Colonoscopy, Detection, Invitation, Prevention, Colorectal cancer screening

## Abstract

**Background:**

Colorectal cancer, a prevalent malignancy worldwide, is associated with numerous modifiable and non-modifiable risk factors that play a role in the early detection and successful treatment of cancer. Despite improvements in the availability and quality of screening methods, especially colonoscopy, and the substantial survival benefits of the early detection of colorectal cancer, patient participation remains low due to clinical reasons and patient barriers. Studies around the world have used various methods of invitation in order to promote patient uptake of colonoscopies. The main objective of this systematic review is to analyze the association between certain invitation procedures, the participation in colonoscopies, and important patient outcomes in the early detection and prevention of colorectal cancer.

**Method:**

We will systematically search in electronic databases including Medline via PubMed and Ovid, Cumulative Index of Nursing and Allied Health Literature (CINAHL), and the Cochrane Library. All studies will be described in a table of study characteristics with a risk of bias assessment. In addition, two authors will independently rate the overall quality of evidence for the critical outcomes using the Grading of Recommendations, Assessment, Development and Evaluation (GRADE) Working Group approach. Discrepancies regarding the inclusion of studies, data extraction, or risk of bias assessment will be resolved independently by one other reviewer. Due to the heterogeneous design of the studies that will be evaluated in this review, synthesizing data from these studies in the form of a meta-analysis may not be possible. In this instance, we can conduct a descriptive synthesis of data from these studies.

**Discussion:**

The results that arise from this systematic review will reflect the influence that various invitation procedures to a colonoscopy have on patient participation in these screenings. Drawing conclusions about the efficiency of various invitation methods to a colonoscopy can provide valuable information to both clinicians and patients and may not only improve future invitation-based patient recruitment to colonoscopy screenings, but also shape guidelines regarding prevention of colorectal cancer.

**Systematic review registration:**

PROSPERO CRD42019128645

## Background

Worldwide, colorectal cancer (CRC) is the third most prevalent malignancy and the fourth most common cause of cancer-associated death, accounting for over 1 million new cancer diagnoses and over 600,000 cancer deaths each year [1, 2]. A great number of factors including dietary and environmental influences, age, gender, ethnicity, race, health habits (tobacco usage and heavy alcohol consumption, physical inactivity, body weight), personal or familial history of cancer or colorectal polyps, history of inflammatory bowel disease, and other syndromes such as the lynch syndrome (HNPCC) or familial adenomatous polyposis (FAP) contribute to the persistent incidence of colorectal cancer in the general population [3, 4].

In the past three decades, CRC survivorship has significantly increased due to improved awareness within the general population and the development of screening methods [3]. According to the US Preventive Services Task Force (USPSTF), CRC screening in adults ages 50 to 75 years with one or more of the various available screening methods can accurately detect early stages of CRC and precancerous lesions and reduce the CRC mortality [5]. Furthermore, early detection of colorectal neoplasms broadens the spectrum of treatment options [3]. Studies done by the USPSTF show a strong correlation between the time of cancer detection and treatment and CRC mortality [5]. For example, the 5-year survival rate for patients diagnosed with CRC in an early stage (local growth) is 90%, reflecting a substantial mortality benefit compared to 71% at the regional stage and 14% at the distant stage of growth [3].

The gold standard for CRC screening and surveillance is the colonoscopy, a highly sensitive method of choice for the detection and removal of CRC and precancerous lesions [6]. Despite clear evidence reflecting the benefits of colonoscopy screening, participation in screening procedures is low due to modifiable and non-modifiable factors [7]. A recently published German study aimed at increasing colonoscopy uptake of the population at familial risk for CRC associated these low participation rates with both clinical and patient-specific factors [6]. Researchers concluded that extensive colon preparation prior to this invasive procedure and a risk of side effects and complications in two out of every 1000 colonoscopies contribute to low patient participation [6]. Additional barriers for patients include unawareness of CRC screening options, procedure-related anxiety, and an inaccurate understanding of such screening methods [6]. Often, misperceptions of family history and the associated familial CRC risk, miscommunication within the family regarding CRC prevention, and other socioeconomic factors can negatively influence patient participation rates [6].

A great number of studies of patient groups around the world have focused on promoting patients’ awareness and understanding of colonoscopy screening in order to increase screening participation and CRC prevention. For example, a study conducted at a primary care clinic in New York City reveals a 20–27% increased rate of referral to a screening colonoscopy when patients were given handouts and/or counseling addressing the barriers they may face and concerns they may have about a colonoscopy [8]. In addition, a wide range of invitation strategies, ranging from mailed invitation letters to education resources (brochures and videos) and activities (media campaigns and counseling) [9], and patient eligibility criteria targeting a variety of patient groups, have been explored in order to achieve higher patient participation. A systematic review published in 2011, for example, aimed to compare patient uptake of various CRC screening methods after the first invitation and also reported on factors affecting the uptake rate such as invitation method, population, and sociodemographic characteristics [10]. However, the review only deemed studies of trials in average-risk populations as eligible, excluding those studies of patients with an elevated familial CRC risk [10]. In a more recent study from 2014 published by researchers in Ontario, Canada, the effectiveness of using physician-linked mailed invitations to increase patient uptake of either a fecal occult blood test (FOBT) or colonoscopy in a cancer screening program was analyzed [11]. The authors found the use of physician-linked mailed invitations to increase screening participation by 14%. However, one acknowledged limitation during patient recruitment was the inability to determine the patients’ family history, including the familial risk of CRC [11].

This systematic review sets out to provide evidence of effective invitation procedures for CRC screening in the average-risk population and population at familial risk for CRC, incorporating the baseline factors of age and gender into the summary of findings. This approach sets it apart from many studies, including ones conducted by the USPSTF, which only include the asymptomatic population and do not specifically focus on the population with familial CRC risk [12]. Understanding the multi-factorial nature of low colonoscopy screening uptake across populations and illustrating how and which different invitational approaches increase patient participation (uptake) in colonoscopies can improve colorectal cancer detection and prevention.

## Methods/design

This protocol of the systematic review adheres to the recommendation of the preferred reporting items for systematic reviews and meta-analysis protocols (PRISMA-P 2015) guidelines (see Additional file 1: PRISMA-P 2015 Checklist) [13]. The protocol of this systematic review is registered in the PROSPERO database, the International prospective register of systematic reviews (CRD42019128645).

### Eligibility criteria

We will use the population, intervention, comparators, and outcomes (PICO) framework for the purpose of this systematic review to clearly define the different components of this review and to aid in study selection. This will allow us to gather relevant information in order to conclusively determine the effectiveness of various CRC screening invitation procedures in the early detection of colorectal cancer.

#### Study design

The database search will include randomized controlled trials (RCT).

#### Participants

We will include studies of patients of all age groups, gender, comorbidities, ethnicities, and geographical areas independent of their CRC risk.

#### Interventions and comparators

The intervention(s) will be any method or combination of methods of invitation to colorectal cancer prevention screening (colonoscopy) participation. These methods can be compared with one another to assess the effectiveness of the uptake rate of (participation in) colorectal cancer prevention screening, specifically a colonoscopy, in regard to the preceding method of invitation.

Studies of interest will feature one or various invitation method(s) to a colonoscopy: stepwise mailed intervention invitation letters [14] with free bowel prep or FIT kits [15], scheduling assistance [15], and reminder calls [15]; advanced notification letters [16]; mailed outreach letters [17]; physician-linked mailed invitation letters [11]; postcards; automated call back systems [18]; specific education and outreach activities (including point-of-care videos and phone lines dedicated to patient education) [19]; physician recommendation; pamphlets/informational brochures about colorectal cancer [9]; or media campaigns (including, but not limited to, online video sharing, widgets, blogs, and social media forums) [20].

#### Outcomes

The primary outcome will be the medically verified patient participation in (uptake of) a colonoscopy, one type of colorectal cancer screening method, following different types of invitation procedures. The secondary outcomes are the neoplasm detection rate, advanced neoplasm findings, CRC incidence, psychological distress due to CRC screening, screening complications, counseling time spent with patients before colonoscopy uptake, and patient barriers against a colonoscopy. We will only include studies reporting on the primary and at least one of the secondary outcomes.

#### Setting

No restrictions in regard to setting/geographical area will be put into place.

#### Further inclusion criteria

The search for studies will be limited to those published in the English or German languages. We will include only full-text publications that have reported methods and findings according to the CONSORT statement [21], as sufficient details are required for assessing the internal validity of included studies.

#### Exclusion criteria

Newspaper articles, editorials, popular media, conference proceedings, and book articles will not be included in the review due to the possibility of publication bias and subjectivity. Articles about studies that fail to meet any of the eligibility criteria listed above will likewise be excluded from the review.

### Information sources and search strategy

We will conduct a systematic search in electronic databases including Medline via PubMed and Ovid, Cumulative Index of Nursing and Allied Health Literature (CINAHL), and the Cochrane Library using a series of search strategies developed on the basis of keywords, study design, timeframe, and language restrictions. A comprehensive overview of the developed search strategy for each database can be found in an online appendix (see Additional file 2: Appendix 1: Draft for the database search strategies). The search for studies will be limited to published studies whose publication dates lie within the last 10 years (2009–2019). Clinical trial registries will be searched in order to identify unpublished studies that may be eligible for inclusion in this review. We will contact study investigators for unpublished full-text reports and include studies if they allow an assessment of the internal validity of the study. This will enhance the study analysis process, as it allows us to draw conclusions about the potential publication bias of completed studies that were not published. In addition, all reference lists of included studies and systematic reviews will be checked for further eligible studies. References of relevant studies will be exported into and organized using the Citavi Reference Manager. A search for eligible studies will be repeated 3 months prior to the completion of this systematic review to include the most recent results of available research. The systematic review is anticipated to be completed in April 2020.

### Study selection

The selection of relevant studies will be ensured by using a 2-stage approach. In phase one, we will pre-screen study titles and abstracts against the eligibility criteria. Papers that clearly do not meet the inclusion criteria defined in this review will be excluded at this stage. In phase two, the full texts of those studies identified as potentially relevant in the first screening will be retrieved and again screened against the eligibility criteria. These steps will be done independently by two authors. Uncertainty regarding study eligibility will be discussed with and, if necessary, resolved by one other reviewer.

A PRISMA-based flow diagram [22] will record and illustrate the transparency of the process of study selection at the different stages, including the exclusion of studies (with a description of the motives for exclusion of those studies omitted upon a review of their full text).

### Data extraction

One author will extract the following information from all included studies and organize relevant data into a data extraction form. The most important results will be organized into a table of study characteristics. All extractions will be checked by another author.

This table will include the following characteristics from each study, when available:

- General information: Title, authors, date of publication, country/geographical area, date of study recruitment, study design (individual patient or cluster-RCT), and sponsorship
- Study design: Sample size and follow-up
- Participants: Inclusion and exclusion criteria, age, gender, family history/familial risk, comorbidities, and ethnicity
- Intervention: Scheme and duration, organization, and type of screening program
- Outcomes: Rate ratios (RRs) and their 95% confidence intervals (CI), number of events and patients per group for uptake of colonoscopy, neoplasm detection rate, advanced neoplasm findings, CRC incidence, psychological distress due to CRC screening, screening complications, mean or median and range of counseling time spent with patients before colonoscopy uptake, and available results on patient barriers against a colonoscopy and psychological burdens.

### Risk of bias assessment

Two authors will independently assess the internal validity of ultimately included studies to identify areas of strength and weakness of the existing evidence. Disagreements will be resolved through discussion. Risk of bias will be described by and judged in seven specific domains according to the Cochrane risk of bias tool, and studies will be placed into one of the three categories, “low,” “high,” or “unclear” [23]:

- Selection bias due to inadequate random sequence generation or allocation concealment (2 domains per study)
- Performance bias due to inadequate blinding of participants and personnel (to be assessed for each critical outcome per study)
- Detection bias due to inadequate blinding of outcome assessment (to be assessed for each critical outcome per study)
- Attrition bias due to incomplete outcome data (to be assessed for each critical outcome per study)
- Reporting bias due to selective reporting (1 domain per study)
- Other bias not covered elsewhere (1 domain per study)

The overall risk of bias will be considered “low” if all seven domains of the risk of bias are rated “low.” In the case that one domain is rated as “high” or “unclear,” the overall risk of bias will be considered “moderate.” A “high” overall risk of bias will be assumed if more than one domain is rated “high” or “unclear.”

The final paper will include a risk-of-bias overview table of all included studies, which will be used to judge the quality of evidence.

Critical outcomes are essential for recommendations in guidelines and include uptake of screening, detection rate of advanced neoplasm, psychological distress due to CRC screening, and screening complications. We will rate the overall quality of evidence of these critical outcomes using the Grading of Recommendations, Assessment, Development and Evaluation (GRADE) Working Group methodology [24].

### Data analyses

The medically verified patient uptake of a colonoscopy, the neoplasm detection rate, advanced neoplasm findings, CRC incidence, complications of screenings, and the psychological distress due to CRC screening of the intervention and control group will be compared on the basis of RR and their 95% CI. A RR > 1 will present a higher rate in the intervention group. Counseling time spent with patients before colonoscopy uptake will be compared with mean differences (MD), with positive differences indicating longer times in the intervention group. The patient barriers against a colonoscopy will be summarized descriptively.

The main analysis of this systematic review will incorporate all studies across different invitation methods with a low or moderate risk of bias. Effect measures for all primary and secondary outcomes will be visually presented with their 95% CI in the form of forest plots. This systematic review will bring together material with an element of diversity differing in participants and interventions studied. Therefore, we do not expect a single study effect and will apply random effects. To quantify the extent of variability among the collection of studies, we will quantify heterogeneity as a proportion of variability *I*^2^ and calculate the between-study variance *τ*^2^ [25, 26].

The dependence of the intervention effect on differences in study characteristics will be explored in additional exploratory subgroup analyses, independent of the results of the heterogeneity tests in order to explore potential effect modifiers. Subgroup analyses of the various invitation methods to colonoscopy will be conducted in order to understand and evaluate their effectiveness. Differences between intervention effects may be influenced by the following study characteristics and will also be investigated in subgroup analyses:

- Differences in the population (average-risk population and population at familial risk, different age groups, and gender)
- Differences in screening programs (grade of organization, cost-coverage, and preferred screening methods)
- Differences in quality (“low” vs. “high” or “unclear” risk of bias) of the studies

Additionally, we will investigate the influence of pre-specified decisions in the process of meta-analysis in sensitivity analyses. Sensitivity analyses are planned for the method of data synthesis (random versus fixed effects model). Other issues will be identified during the review process, when the individual peculiarities of the studies under investigation are identified. If and when these analyses identify particular decisions that greatly influence the findings of the review, the results must be interpreted with an appropriate degree of caution. Furthermore, funnel plots will be used to investigate the unexplained asymmetry of effect sizes due to small study effects. These effects might be caused by different reasons including clinical or methodological heterogeneity and potential publication bias.

#### Secondary endpoints

All outcomes which cannot be presented in forest plots will be analyzed descriptively. Statistical analysis will be performed using Review Manager. In the instance that statistical pooling is not possible, the findings will be presented in narrative form.

### Assessment of the overall quality of the evidence

Two authors will independently rate the overall quality of the evidence for the critical outcomes (uptake of screening, detection rate of advanced neoplasm, psychological distress due to CRC screening, and screening complications) according to the Grading of Recommendations, Assessment, Development and Evaluation Working Group (GRADE) approach [27, 28]. Each critical outcome’s quality of evidence is rated, taking into consideration five criteria (risk of bias and limitations of design, consistency of analyzed studies and their results, directness, precision, and publication bias) that may lead to grading down and three criteria (large effect, dose-response, and opposing bias and confounders) that may lead to grading up [27, 28]. For each comparison, two review authors will independently rate the quality of evidence for the critical outcomes as “high,” “moderate,” “low,” or “very low” [24]. Disagreements will be resolved by consensus or, if needed, through arbitration by a third review author. The final paper will include a summary of findings table for each comparison including all critical outcomes and important sources of heterogeneity with detailed explanations for the upgrading and downgrading of the quality of evidence. Discrepancies between the two reviewers regarding judgment of the quality of evidence will be resolved either through discussion or by incorporating a third reviewer into the review process.

## Discussion

Colorectal cancer is a growing concern worldwide and interventions focused on increasing awareness and screening participation are necessary. The various kinds of invitation procedures to colonoscopy screening are recognized as key factors in promoting patient uptake of CRC screening and thus contribute towards early detection and prevention of this malignancy. This systematic review aims to identify which invitation procedures to a colonoscopy screening are more effective than others at improving colorectal cancer detection and prevention, taking into consideration the patients’ CRC risk along with the baseline factors age and gender.

In this systematic review, the study protocol, reflecting a search strategy based on the keywords “colorectal neoplasm” and “early detection of cancer” and “colonoscopy” and “randomized controlled trial” and plan of data extraction and analysis along with an in-depth quality assessment based on the GRADE approach are major strengths. The main analysis of this systematic review will incorporate only studies with a low or moderate bias risk. We will exclude studies with a high risk of bias, as these studies may influence the precision of this review, presenting a potential problem. A limitation related to the scope of this review is that it only includes colonoscopy, which is just one of several CRC screening methods currently being used. Lastly, publication bias could affect results, as the predetermined language limitation will restrict analysis of relevant studies to those studies published in the English or German language. The results from this systematic review will detail the influence that various invitation procedures to a colonoscopy have on patient participation in these screenings. Summarizing the effectiveness of various invitation procedures to colonoscopy screening can provide patients and clinicians with valuable information to improve patient treatment and decision-making. In the future, these results may influence invitation-based patient recruitment to colonoscopy screenings and play a role in shaping colorectal cancer detection and prevention guidelines.

## Supplementary information

**Additional file 1:.** PRISMA-P 2015 checklist

**Additional file 2:.** Appendix 1: Draft for the database search strategies

## Data Availability

Not applicable.

## Abbreviations

CRC: Colorectal cancer
HNPCC: Hereditary nonpolyposis colorectal cancer or Lynch syndrome
FAP: Familial adenomatous polyposis
USPSTF: US Preventive Services Task Force
FOBT: Fecal occult blood test
PICO: Population, intervention, comparators, and outcomes
RCT: Randomized controlled trial
PRISMA: Preferred Reporting Items for Systematic Reviews and Meta-Analyses
GRADE: Grading of Recommendations, Assessment, Development and Evaluation
CINAHL: Cumulative Index of Nursing and Allied Health Literature
RR: Rate ratio
CI: Confidence interval
MD: Mean difference
