# Supplementary material for: What is the effectiveness of various invitation methods to a colonoscopy in the early detection and prevention of colorectal cancer? Protocol of a systematic review
Source: Syst Rev. 2020 Mar 6;9:49. doi: 10.1186/s13643-020-01312-x (PMC7059336; doi:10.1186/s13643-020-01312-x)
Supplement: Supplementary file 2 — Additional file 2:. Appendix 1: Draft for the database search strategies [file 13643_2020_1312_MOESM2_ESM.docx]

**Additional File 2: Appendix 1: Draft for the database search strategies**

**PubMed**

| Search | Add to builder | Query | Items found | Time |
| --- | --- | --- | --- | --- |
| [#24](https://www.ncbi.nlm.nih.gov/pubmed/advanced) | [Add](https://www.ncbi.nlm.nih.gov/pubmed/advanced) | Search **(((((((colorectal cancer) OR colorectal neoplasms) OR "Colorectal Neoplasms"[Mesh])) OR early detection of cancer) OR "Early Detection of Cancer"[Mesh])) AND colonoscopy** Filters: **Randomized Controlled Trial; published in the last 10 years; English; German** | [529](https://www.ncbi.nlm.nih.gov/pubmed/?cmd=HistorySearch&querykey=24) | 00:04:39 |
| [#23](https://www.ncbi.nlm.nih.gov/pubmed/advanced) | [Add](https://www.ncbi.nlm.nih.gov/pubmed/advanced) | Search **(((((((colorectal cancer) OR colorectal neoplasms) OR "Colorectal Neoplasms"[Mesh])) OR early detection of cancer) OR "Early Detection of Cancer"[Mesh])) AND colonoscopy** Filters: **Randomized Controlled Trial; published in the last 10 years; English** | [529](https://www.ncbi.nlm.nih.gov/pubmed/?cmd=HistorySearch&querykey=23) | 00:04:29 |
| [#22](https://www.ncbi.nlm.nih.gov/pubmed/advanced) | [Add](https://www.ncbi.nlm.nih.gov/pubmed/advanced) | Search **(((((((colorectal cancer) OR colorectal neoplasms) OR "Colorectal Neoplasms"[Mesh])) OR early detection of cancer) OR "Early Detection of Cancer"[Mesh])) AND colonoscopy** Filters: **Randomized Controlled Trial; published in the last 10 years** | [534](https://www.ncbi.nlm.nih.gov/pubmed/?cmd=HistorySearch&querykey=22) | 00:04:16 |
| [#21](https://www.ncbi.nlm.nih.gov/pubmed/advanced) | [Add](https://www.ncbi.nlm.nih.gov/pubmed/advanced) | Search **(((((((colorectal cancer) OR colorectal neoplasms) OR "Colorectal Neoplasms"[Mesh])) OR early detection of cancer) OR "Early Detection of Cancer"[Mesh])) AND colonoscopy** Filters: **published in the last 10 years** | [10155](https://www.ncbi.nlm.nih.gov/pubmed/?cmd=HistorySearch&querykey=21) | 00:04:13 |
| [#19](https://www.ncbi.nlm.nih.gov/pubmed/advanced) | [Add](https://www.ncbi.nlm.nih.gov/pubmed/advanced) | Search **(((((colorectal cancer) OR colorectal neoplasms) OR "Colorectal Neoplasms"[Mesh])) OR early detection of cancer) OR "Early Detection of Cancer"[Mesh]** | [272758](https://www.ncbi.nlm.nih.gov/pubmed/?cmd=HistorySearch&querykey=19) | 00:03:04 |
| [#18](https://www.ncbi.nlm.nih.gov/pubmed/advanced) | [Add](https://www.ncbi.nlm.nih.gov/pubmed/advanced) | Search **((colorectal cancer) OR colorectal neoplasms) OR "Colorectal Neoplasms"[Mesh]** | [228419](https://www.ncbi.nlm.nih.gov/pubmed/?cmd=HistorySearch&querykey=18) | 00:02:29 |

**Medline via Ovid**

| **#** | **Searches** | **Hits** |
| --- | --- | --- |
| 1 | *Colorectal Neoplasms/ | 71 151 |
| 2 | colorectal neoplasm*.ti,ab. | 1331 |
| 3 | colorectal cancer.ti,ab. | 81 158 |
| 4 | 1 or 2 or 3 | 100 988 |
| 5 | *Colonoscopy/ | 12 009 |
| 6 | colonoscopy.ti,ab. | 22 210 |
| 7 | early detection of cancer.ti,ab. | 1062 |
| 8 | 5 or 6 or 7 | 27 261 |
| 9 | randomized controlled trial.pt. | 483 981 |
| 10 | controlled clinical trial.pt. | 93 102 |
| 11 | randomized.ab | 407 947 |
| 12 | randomly.ab. | 280 745 |
| 13 | trial.ab | 727 475 |
| 14 | 9 or 10 or 11 or 12 or 13 | 1 109 530 |
| 15 | 4 and 8 and 14 | 1097 |
| 16 | exp animals/ not humans/ | 4 591 519 |
| 17 | 15 not 16 | 1097 |
| 18 | limit 17 to yr="2009 -Current | 743 |

**CINAHL**

TI (colonoscopy or early detection) AND TI (randomised or randomized) AND TX (neoplasm OR cancer )

120 Results

**CENTRAL**

| **#** | **Searches** | **Hits** |
| --- | --- | --- |
| 1 | MeSH descriptor: [Colorectal Neoplasms] explode all trees | 7213 |
| 2 | colorectal neoplasm* | 7128 |
| 3 | colorectal cancer* | 14744 |
| 4 | #1 or #2 or #3 | 17147 |
| 5 | MeSH descriptor: [Colonoscopy] explode all trees | 1891 |
| 6 | colonoscop* | 5994 |
| 7 | early detection of cancer | 3657 |
| 8 | #5 or #6 or #7 | 9477 |
| 9 | #4 and #8 with Cochrane Library publication date from Jan 2009 to Jul 2019, in Trials | 2076 |
| 10 | invitation | 1390 |
| 11 | #9 and #10 | 106 |
